# Hearing Loss Detection and Early Intervention Strategies in Kenya

**DOI:** 10.5334/aogh.4336

**Published:** 2024-02-05

**Authors:** Serah Ndegwa, Michelle Pavlik, Emily R. Gallagher, Maureen King’e, Manaseh Bocha, Lilian Wairimu Mokoh, Isaac Macharia, Paige Stringer, Irene Njuguna, Dalton Wamalwa, Sarah Benki-Nugent

**Affiliations:** 1 Department of Surgery, University of Nairobi, Nairobi, Kenya; 2 Kenyatta National Hospital, Nairobi, Kenya; 3 Department of Health Systems and Population Health, University of Washington, Seattle, WA, USA; 4 Departments of Pediatrics and Global Health, University of Washington, Seattle, WA, USA; 5 Seattle Children’s Hospital, Seattle, WA, USA; 6 Department of Paediatrics and Child Health, University of Nairobi, Nairobi, Kenya; 7 Clinical Services, Ministry of Health, Nairobi, Kenya; 8 Kenyatta University Teaching, Referral and Research Hospital, Nairobi, Kenya; 9 Global Foundation For Children With Hearing Loss, Poulsbo, WA, USA; 10 Department of Global Health, University of Washington, Seattle, WA, USA

**Keywords:** hearing screening, hearing loss, pediatrics, neurodevelopment

## Abstract

**Background:**

Thirty-four million children globally have disabling hearing loss, with the highest prevalence in low- and middle-income countries (LMICs). Early identification and management is crucial, yet barriers to screening and treatment of hearing loss are extensive in LMICs. Unaddressed hearing loss negatively impacts individuals and communities. The WHO’s 2021 World Report on Hearing urges the development of Ear and Hearing Care (EHC) programs to improve access to all aspects of care, including screening, diagnostics, management, and developmental support. A joint Nairobi- and Seattle-based group convened in 2021 to discuss strategies for program development in Kenya, as presented in this paper.

**Findings:**

Developing a national EHC program must include the necessary support services for a child with a diagnosed hearing loss, while simultaneously promoting engagement of family, community, and healthcare workers. Existing government and healthcare system policies and priorities can be leveraged for EHC programming. Strategies for success include strengthening connections between policymakers at national, county, and municipal levels and local champions for the EHC agenda, with a concurrent focus on policy, early detection and intervention, habilitation, and family-centered care. Updates to health policy and funding to support the accessibility of services and equipment should focus on leveraging national healthcare coverage for hearing technologies and services, strengthening referral pathways, training to bolster the workforce, and metrics for monitoring and evaluation. Additional strategies to support forward progress include strategic engagement of partners and leveraging local partners for phased scale-up.

**Conclusions and Recommendations:**

Recommendations to strengthen EHC within the Kenyan health system include concurrent leverage of existing health policies and priorities, partner engagement, and strengthening referral pathways, monitoring and evaluation, and training. These strategies may be generalized to other countries too.

## Introduction

Globally, approximately 34 million children have disabling hearing loss, defined as moderate or worse hearing (35 dB) in the better-hearing ear [1]. The percentage of children with hearing loss in low- and middle-income countries (LMICs) is more than double compared with high-income countries (1% vs. 0.4%, respectively, and possibly as high as 3% in Africa) [2,3]. In Kenya, 14 in every 1,000 children have moderate or severe hearing loss, which is around 10 times higher than in the United States [4,5]. Risk factors for acquired hearing loss include exposures that are more common in LMICs, such as congenital infections (e.g., toxoplasmosis, rubella, cytomegalovirus, and herpes) [6], premature birth [7], in utero exposure to HIV [8], neonatal jaundice [9], recurrent ear infections [10,11], HIV infection [12], bacterial meningitis, and ototoxic medications such as those used to treat tuberculosis [13], pneumonia [14], or malaria [15]. The WHO World Report on Hearing indicates that 40% of hearing loss is unavoidable, however early intervention may mitigate impacts [14].

Identification and treatment of hearing loss typically occurs late in LMICs [17]. Late detection or deferred interventions can have a profound impact on speech and language skills, academic achievement, and social development [16,18,19]. Infants with hearing loss detected after 6 months of age have delayed language scores regardless of cognitive abilities, degree of hearing loss, or other confounding factors [14], whereas children with hearing loss that are detected and managed before 6 months of age are more likely to have typical development [16]. Not surprisingly, unaddressed hearing loss negatively impacts the economic status of individuals, communities and countries, with an additional global cost of 980 billion international dollars (IDs) per year [16]. WHO estimates the return for universal newborn hearing screening (UNHS) in LMICs is 1.67 IDs for every 1 ID invested [16]. Providing cochlear implants or hearing aids can save 1.46–1.62 IDs for every 1 ID invested within an LMIC [16]. In India, UNHS saved over 500,000 IDs per infant identified [20]. However, lack of adequate human resources and high cost of hearing screening and diagnostic equipment in LMICs, pose critical barriers to these benefits. For example, a cost study in Kenya and five other sub-Saharan African countries found that deaf education was cost-effective and that cochlear implants could be cost-effective if devices were priced relative to gross domestic product [21].

The WHO urges all WHO Member States and international partners to establish training programs to increase EHC human resource development, improve access to affordable EHC equipment, and integrate EHC promotion strategies into the framework of their primary healthcare system [16]. The WHO recognizes Kenya as a reference point for other countries in the East and Central African region in the development of a national strategic plan for EHC [16]. Kenya launched its initial 5-year EHC national strategic plan in 2016, and has continued efforts in response to WHO guidelines [16]. The national strategic plan called for measures such as pathways for EHC professionals to enter public service, providing coverage for hearing aid costs and cochlear implants through the National Hospital Insurance Fund, increasing resources for training for EHC workers, and improving the infrastructure of EHC facilities [16]. However, this national strategic plan was not fully implemented as envisioned, in part because there was no appointed focal person within the Ministry of Health to drive the implementation process of the strategic plan, or terms of reference to guide implementation and monitoring.

The Ministry of Health of Kenya has prioritized Universal Health Coverage (UHC), in keeping with the WHO 2030 Sustainable Development Goal 3.8: “Achieve universal health coverage, including financial risk protection, access to quality essential health care services, and access to safe, effective, quality, and affordable essential medicines and vaccines for all [22,23].” The Kenyan Health Policy (2014–2030) likewise focuses on increasing access and coverage of all aspects of healthcare, and ensuring financial protection grounded on a National Health Insurance Fund [24], hence national policies are in place to support EHC implementation. Healthcare in Kenya is directed by the Ministry of Health, while the provision of healthcare services within the public system is devolved to county governments. The national government provides policies, standards, and technical guidelines to its 47 counties, which in turn allocate financial resources for healthcare and determine service provision [23]. In addition, the majority of the Kenyan population resides in rural areas (72% as of 2021) [25], necessitating robust EHC referral pathways from smaller facilities in rural areas to larger facilities and hospitals in urban areas.

Considering the negative impact of unaddressed hearing loss in children, the high prevalence of childhood hearing loss in Kenya, and the recent global push for improved EHC services [16], a joint Nairobi- and Seattle-based group gathered stakeholders to discuss options for increased EHC services, with particular emphasis on programming that included UNHS for early hearing loss identification and care provision during the crucial developmental window of 6 months following birth. This paper summarizes key strategies for program strengthening identified during the workshop. These strategies may be adapted to benefit other LMICs that wish to strengthen their EHC programs.

## Gathering Perspectives from Stakeholders

**Figure 1:**
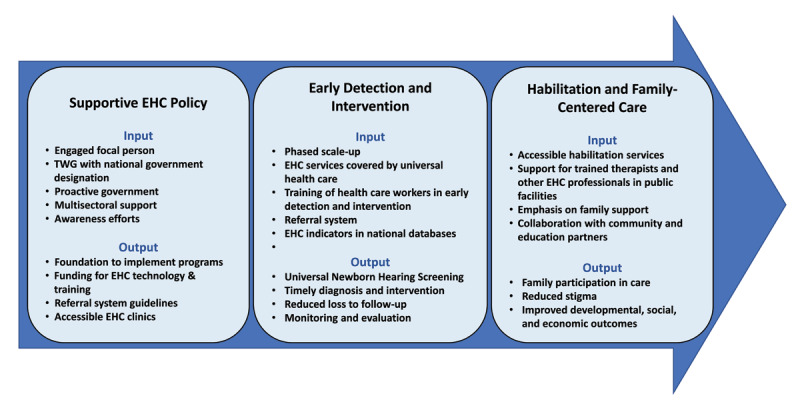
Inputs and outputs in successful ECH programming. *Note*: EHC, ear and hearing care; TWG, technical working group.

Clinicians, researchers, policymakers (from academic institutions and the Ministry of Health), and advocates with interest or involvement in the ear and hearing care services were invited to a workshop in Nairobi, Kenya. Discussion focused on early detection and intervention, requisite services for children diagnosed with hearing loss, human resource needs, and next steps. Speakers discussed global best practice methods, recent recommendations from the 2021 WHO World Report on Hearing, and current EHC services in Kenya. Participants conducted a Strengths, Weaknesses, Opportunities, and Threats (SWOT) analysis of EHC in Kenya and identified strategies to address priorities, which included improving early hearing detection and diagnostic evaluation, developmental support, and habilitation services, providing training for more EHC professionals to improve equitable access to EHC and habilitation services countrywide, and implementing the national strategic plan for EHC. Here we present summaries from this discussion, together with generalizable policy inputs and other strategies for programmatic progress on EHC that are tailored for LMICs (Figure 1).

## Key Themes and Strategies

### Early hearing detection and intervention

#### Context

The Joint Committee on Infant Hearing, with representation from the American Academy of Pediatrics, American Academy of Otolaryngology-Head and Neck Surgery, and others, recommends adopting the Early Hearing Detection and Intervention (EHDI) 1-3-6 goals [26]. These include an initial hearing screen completed by 1 month of age, diagnosis by 3 months for a child who does not pass the initial screen, and enrollment in early intervention by 6 months for a child diagnosed with hearing loss. Once identified, interventions include medical and surgical management for related conditions and habilitation for those with irreversible hearing loss. UNHS significantly lowers the average age at which a child is identified with hearing loss, and adherence to the 1-3-6 goals maximizes language and communication competence, literacy development, and psychosocial well-being for children who are deaf or hard of hearing [26]. In addition to UNHS, it is important to detect late-onset hearing loss in preschool and school-aged children.

In LMICs, UNHS and school-based hearing screening programs remain inadequate. Nearly all countries in Africa screen 0%–1% of newborns and infants, with only South Africa reporting that 1%–9% are screened in a recent global study of UNHS [27]. Among the barriers for EHC programs are human resource gaps and access to equipment for hearing screening and diagnosis [28], which are cost-prohibitive in most LMICs. Otoacoustic Emissions (OAE) or Automated Auditory Brainstem Response (AABR) detection devices are typically used for newborn hearing screening and can cost $6,000–$10,000 USD [16]. Diagnostic equipment needed to confirm a diagnosis is more costly and requires more training to administer. Some current mobile-based technologies (Shoebox™, HearTest™, and hearWHO) [29,30,31] are lower in cost but are not designed for hearing assessment of infants and young children.

#### Strategies

Kenya is poised for UNHS in hospitals and in well-baby clinics due to high facility-based births at 82% (covered by its national health insurance plan) and high (80%) infant vaccination rates [32]. However, high cost and poor availability of screening and diagnostic equipment, limited clinic space, limited availability and use of health information systems, a limited number of trained healthcare workers and available services in habilitation (e.g., pediatric audiology, auditory verbal therapy, and early intervention), and overburdened staff are barriers. In addition, limited awareness among both parents and healthcare workers about EHC and the importance of early interventions, the lower perceived value of hearing screening, high cost of screening, and access to technology and habilitation, could each hinder acceptance and feasibility. Importantly, the Kenya Ministry of Health Mother & Child Health Handbook, both a key resource for health information for parents, and a health service utilization and health documentation tool, lacks prompts to address caregiver concerns related to hearing and to document hearing screening and diagnostic evaluation results [33,34]. Given these structural issues, government support, policy creation, and resource allocation are each critical steps toward the successful implementation of UNHS.

A strategic, phased scale-up approach beginning in two to three exemplar counties with support from local policymakers and EHC champions could be used to strategically launch pilot UNHS programs in concert with the multiple requisite services for follow-up. Such programs would ideally be situated in select hospitals located in different geographic regions and equipped with requisite screening and diagnostic equipment, dedicated space, procedures for documenting screening results and tracking referrals, and trained staff to provide necessary follow-up services. The inclusion of services for diagnostic evaluation and interventions under UHC would provide families with the necessary support in attaining these services. Strengthening the number of healthcare workers trained to provide early detection and refer for intervention services would also be important for the ultimate success of scaled-up hearing screening in Kenya.

In addition to early identification, timely access to hearing technology and cochlear implants, habilitation services and family engagement are required to achieve optimal outcomes [35]. Facilities equipped to conduct hearing screening would therefore ideally have access or referral pathways to affordable hearing technology (e.g., cochlear implants and hearing aids) for the management of hearing loss and necessary services in audiology, auditory verbal therapy, and early intervention to promote development.

Support must move beyond the specific guidelines for screening and diagnosis to include appropriate scopes of practice for each healthcare cadre at all levels of healthcare facilities. Referral pathways must be developed to provide a clear transition from screening to diagnosis to treatment. The referral pathway should also include medical and surgical management including for preventative hearing loss. Metrics for these steps could be incorporated into the District Health Information System version 2 (DHIS2), currently deployed by 76 countries on a national scale, including Kenya [36]. Modifying DHIS2 in Kenya to include these aspects of EHC will allow for ongoing program evaluation from local to national levels. Altogether these strategies could have collateral benefits in promoting family and community awareness and building broader demand for UNHS in other regions throughout Kenya. Additionally, a forward-looking strategy would be to develop affordable diagnostic technology that is simple to use to further promote EHC.

### Habilitation and family-centered care

#### Context

Identification of children with hearing loss is recommended only if management options are concurrently available. Families often struggle financially and procedurally to coordinate services for their children, especially where EHC services are sparse [16]. Individualized, family-centered guidance and resources improve support for parents and caregivers, as do community groups [16]. Access to hearing technology is challenging in many LMICs, with caregivers required to pay for hearing aids or cochlear implants independently. After acquiring hearing technology, patients need ongoing access to trained providers for appropriate hearing aid fitting and support. Interwoven and multifaceted services are essential to help children with hearing loss reach their full potential, communicate with spoken language, attend mainstream schools, and pursue careers [35]. Children who have timely and full access to hearing technology and developmental support early in life are able to learn to listen and talk and will be less likely to need ongoing accommodations in mainstream schools. In addition, education systems have a critical need to train educators to accommodate children with deafness who use sign language, whether in schools specifically for children with deafness or in integrated classrooms. An effective EHC program should address each of these needs.

#### Strategies

Concurrent establishment of EHC policy, early detection and intervention, habilitation, and family engagement are recommended for a successful EHC strategic plan (Figure 1). Access to hearing screening and diagnostic equipment and trained EHC healthcare providers, and a referral system that includes habilitation and family engagement are each important for sustainable EHC programs. At the county level, established tiers of healthcare delivery in the country facilitate the implementation of national goals at local levels, and counties may highlight their own priorities within the national framework. At the same time, national government investment to support broad access to screening and diagnostic services, hearing technology, and habilitation services is still vital. Including services for both habilitation (development of skills a child has yet to develop) and rehabilitation (regaining skills lost because of a disability) services under UHC, in addition to diagnostic evaluation and intervention would help close the gap on access to timely, necessary care following diagnosis of hearing loss, and could provide much needed financial support for families in seeking care.

Improving access to early intervention and education, as well as job training and placement, for children with hearing loss, beginning during infancy and continuing throughout school, would benefit from support through the Ministry of Education and partnering habilitative organizations. Further expansion of community and family support structures would strengthen options for habilitation following diagnosis and could additionally help reduce the stigma surrounding hearing loss. A byproduct of boosted support structures would also raise awareness in the community and potentially lead to improvements in human resource gaps.

### Human resource gaps

#### Context

In general, LMICs have human resource gaps for many aspects of EHC [28]. A 2009–2015 survey reported 0.163 Ear Nose and Throat surgeons, 0.015 audiologists, and 0.034 speech therapists per 100,000, respectively, in Kenya compared with the United Kingdom, where there were 1.0, 4.1, and 16.4 per 100,000 for the same cadres [37,38,39]. In addition to the evaluation of hearing loss, trained EHC providers must also identify potentially reversible conditions affecting hearing, uncover associated medical disorders that can impact an infant’s overall health, and identify conditions that could interfere with modes of communication. Following hearing loss diagnosis, appropriate fitting of hearing technology and referral to habilitation services is essential for improving developmental outcomes. Without sufficient providers with training to address these diverse needs, the ability to minimize the permanent impact of hearing loss on development is limited. Moreover, there is a need to address the ability of mainstream schools to accommodate the needs of children with deafness that have not been addressed [28].

#### Strategies

The current paucity of EHC providers in Kenya is a barrier for UNHS, as well as other aspects of care for children with hearing loss. In addition to ENT surgeons, ENT clinical officers, audiologists, audiology technicians, and speech and language therapists are needed to fully support EHC in Kenya. The scarcity of personnel could be alleviated with a training model that includes community health workers and other lay people who may be trained in certain EHC tasks (such as hearing screening, providing developmental support, hearing aid maintenance, basic audiology tests, and assistance in achieving therapy goals), reserving access to more highly trained specialists when necessary. Community health workers may be ideally positioned to engage families in care. In addition, increasing awareness of EHC may inspire healthcare workers to seek training in EHC, enhancing the number of available specialists. Engaging private sector EHC workers to participate in national EHC programs will strengthen collaborations to promote such training. County-level governments should be involved to improve the sustainability of this training. Supporting local county EHC programs and phased scale-ups could help create opportunities for local governments to tailor programs for both rural and urban settings and recruit requisite personnel.

Limited access to resources, trained educators, and high training costs challenge the expansion of the EHC workforce. Development of sustainable worker education systems using a “train the trainer” model has been successfully used to improve the stream of skilled providers who can teach pediatric audiology and auditory verbal therapy and promote the establishment of the Continuum of Care for infants and young children with hearing loss and their families in Mongolia, Vietnam, and Nepal [40]. This example of an effective partnership between governments and non-governmental organizations can be a model for efficiently expanding human resources while prioritizing sustainability. Establishing systems to promote EHC resource allocation (e.g., training of healthcare workers and designating workspaces) in tandem with existing governmental programs could also improve the sustainability of these efforts. Moreover, investment in early identification and intervention for children with hearing loss reduces the future economic burden by the state to provide additional support at school since a child with early access to interventions will be successful in a mainstream school.

## Workshop Outcomes

**Figure 2:**
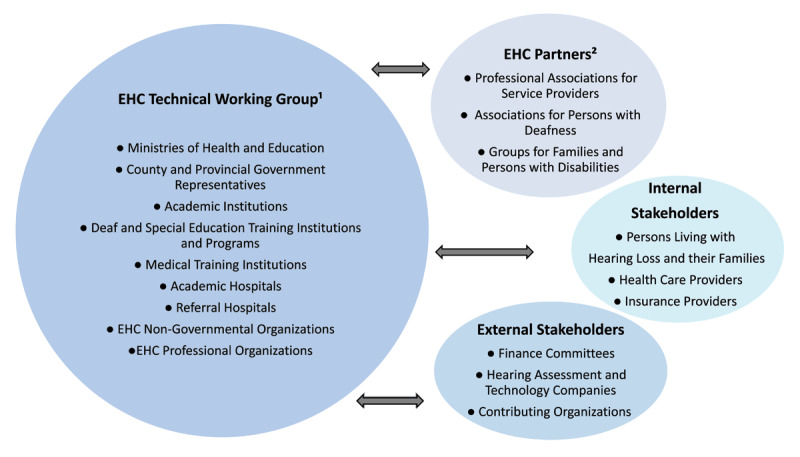
Ear and hearing care multi-sectoral partners. ^1^ Association of Speech and Language Therapists Kenya, Christian Blind Mission (CBM), Council of Governors, Kenya Ear Nose and Throat (ENT) Society, Kenya Institute of Special Education, Kenya Medical Training College, Kenya Society of Audiology, Kenyatta National Hospital, Operation Eardrop Kenya, University of Nairobi. ^2^ Deaf Empowerment Society of Kenya, Association of Speech and Language Therapists Kenya, Kenya ENT Society, Kenya National Association of the Deaf, Kenya Society for Deaf Children, National Council for Persons with Disabilities. *Note*: EHC, Ear and Hearing Care.

Following the workshop, the Ministry of Health (MOH) designated the Technical Working Group (TWG) for EHC. This designation brings support from the Ministry, as well as an expectation that the TWG will be accountable for drafting and implementing strategic planning for EHC. The TWG comprises multi-sector stakeholders and individuals drawn from the Kenya Ministry of Health, the Ministry of Education, the Council of Governors, academic institutions, teaching and referral hospitals, professional associations, and non-governmental organizations (Figure 2), and has identified EHC partners and internal and external stakeholders who can provide valuable guidance and input on strategic planning and implementation. With support from these collaborators, the TWG developed and launched a national strategic plan for EHC care for 2023–2028, which is based on the lessons learned from the attempt to implement the 2016–2019 strategic plan and situational analyses of EHC services in Kenya, including Workshop discussions described here. The TWG has created terms of reference to guide the implementation of the strategic plan and protocols in the health sector. The TWG will continue to follow metrics for monitoring and evaluation to appropriately support other areas of EHC, particularly as they scale up programs from referral hospitals to county and municipal facilities.

The objectives of the Kenyan National EHC strategic plan are to improve access to EHC services, to strengthen the delivery of EHC technologies, to strengthen habilitation and rehabilitation services, and to develop a monitoring and evaluation framework including key indicators for evidence-based decision-making. Identifying EHC stakeholders from regions throughout Kenya with the diversity of professional backgrounds will improve county-level alignment of goals, as well as implementation. Kenya also has training institutions throughout the country to improve capacity for therapists, educators, and healthcare workers focused on EHC. However, Kenya, like many other LMICs, has competing needs, including high poverty and rates of communicable diseases, such as HIV and malaria. These needs require government focus on programs to improve access to food and shelter, as well as other basic needs. The additional economic disparity among regions in Kenya presents the potential for disparate priorities at the county level. However, the designation by the Ministry of Health of a diverse TWG for EHC ensures representation throughout the country and awareness of the risk factors of these exposures on hearing loss throughout the life course.

## Conclusion

Untreated hearing loss can lead to disabling outcomes that are otherwise modifiable if treated early. Current challenges to addressing hearing loss in Kenya include the lack of affordable and accessible screening and diagnostic equipment, limited EHC healthcare workers and trained professionals, and the absence of a well-supported EHC national program. Strengths include partners that are primed to support EHC services and collaborate with the Ministry of Health in resource and training development, a passionate and effective Technical Working Group, an opportune policy window, and a Ministry of Health that is prioritizing EHC programming. We believe many of these lessons learned are applicable to other LMICs.

Unlike some interventions, ear and hearing healthcare is extremely multifaceted. The absence of any aspect of this care is a disservice to patients, families, and partnering groups and could render investments ineffective. The TWG for EHC in Kenya chose to support a holistic program, including UNHS and the range of services required by children diagnosed with hearing loss. To increase the likelihood of successful program implementation, input from invested parties (e.g., county-level governments, families of children with hearing loss, and healthcare workers) should be prioritized. Screening and diagnostic services should be scaled up concurrent with resources for appropriate care (e.g., access to hearing technology, family education, and habilitation services and personnel). The collaboration and development of a comprehensive, multi-sectoral national EHC program is needed to achieve the ultimate goal that all children with hearing loss will have an equitable opportunity to reach their full potential.
